# Preservation of circadian rhythms by the protein folding chaperone, BiP

**DOI:** 10.1096/fj.201802366RR

**Published:** 2019-03-19

**Authors:** Adam Pickard, Joan Chang, Nissrin Alachkar, Ben Calverley, Richa Garva, Peter Arvan, Qing-Jun Meng, Karl E. Kadler

**Affiliations:** * Wellcome Centre for Cell-Matrix Research, Faculty of Biology, Medicine, and Health, Manchester Academic Health Science Centre, University of Manchester, Manchester, United Kingdom;; † School of Mathematics, Faculty of Science and Engineering, University of Manchester, Manchester, United Kingdom;; ‡ Division of Metabolism, Endocrinology, and Diabetes, University of Michigan, Ann Arbor, Michigan, USA

**Keywords:** 4PBA, collagen, ER stress, Per2::luc, UDCA

## Abstract

Dysregulation of collagen synthesis is associated with disease progression in cancer and fibrosis. Collagen synthesis is coordinated with the circadian clock, which in cancer cells is, curiously, deregulated by endoplasmic reticulum (ER) stress. We hypothesized interplay between circadian rhythm, collagen synthesis, and ER stress in normal cells. Here we show that fibroblasts with ER stress lack circadian rhythms in gene expression upon clock-synchronizing time cues. Overexpression of binding immunoglobulin protein (BiP) or treatment with chemical chaperones strengthens the oscillation amplitude of circadian rhythms. The significance of these findings was explored in tendon, where we showed that BiP expression is ramped preemptively prior to a surge in collagen synthesis at night, thereby preventing protein misfolding and ER stress. In turn, this forestalls activation of the unfolded protein response in order for circadian rhythms to be maintained. Thus, targeting ER stress could be used to modulate circadian rhythm and restore collagen homeostasis in disease.—Pickard, A., Chang, J., Alachkar, N., Calverley, B., Garva, R., Arvan, P., Meng, Q.-J., Kadler, K. E. Preservation of circadian rhythms by the protein folding chaperone, BiP.

Circadian clocks are cell-autonomous timekeeping mechanisms that occur in most tissues to optimize cellular activities in anticipation of varying demands on the cell during 24 h (1, 2). One such function is control of translation and protein synthesis (3). In a recent study, Yeung *et al.* (4) showed that the circadian clock regulates translocon-dependent endoplasmic reticulum (ER) protein synthesis and membrane trafficking at each node in the secretory pathway of tendon fibroblasts *in vivo*. The consequence is a daily surge of type I collagen (collagen-I) production, which occurs at night in mice. Collagen-I is the major secreted protein of fibroblasts and, in tendon, accounts for 70% of the mass of the tissue, where it occurs as extracellular fibrils (5) that can extend the length of the tissue (6). Collagens are large trimeric molecules that undergo extensive post-translational modification (7, 8) and folding in the ER (their site of synthesis) in order to generate a thermally stable triple helix capable of assembling into fibrils. The extensive nature of post-translational modification of collagen, coupled with a surge in synthesis at night, suggested that the cell might have mechanisms to protect against the accumulation of unfolded collagen [*e.g.*, with the unfolded protein response (UPR)] and subsequent ER stress at times of high collagen synthesis. Excessive collagen synthesis occurs in fibrosis and solid tumors, where the rate of synthesis exceeds turnover. The result is accumulation of a dense, stiff extracellular matrix that can be a positive fibro-proliferative feedback signal to accelerate cell division and the synthesis of additional matrix (9).

Collagen-I molecules are composed of 2 α1 chains and 1 α2 chain encoded by the genes collagen-I α1 chain (*COL1A1*) and collagen-Iα2 chain (*COL1A2*), respectively. These chains come together to form a triple helix, which is assisted by chaperone proteins. Both generic protein chaperones, such as binding immunoglobulin protein [BiP, also known as heat shock protein (Hsp)A5] or 78-kDa glucose-regulated protein (Grp78) and Grp94, and the collagen-specific chaperone, Hsp47, aid the formation and stabilization of the triple helix (10). Chaperones also have signaling roles associated with their ability to bind newly synthesized proteins. In particular, BiP is known to govern the activation of the UPR (11). When unfolded proteins accumulate in the ER, BiP is released from 3 key binding partners on the ER membrane: protein kinase R–like ER kinase (PERK), inositol-requiring enzyme 1 (IRE1), and activating transcription factor (ATF)6 each activate mechanistically distinct pathways. Combined, the response acts to reduce ER burden through mRNA decay and reducing translation but also drives expression of protein chaperones. However, if the stress is not resolved, these same pathways can also promote apoptosis (12). It has been established that the response to ER stress is regulated in a circadian manner in cancer cells (13) and that activation of UPR in cancer cells induces a 10 h shift in circadian oscillation (14); however, it remains unknown how protein flux through the ER, and in turn ER stress, regulates circadian rhythm in nontumorigenic models. Given that collagen secretion is ramped up from low levels during the day to high levels in the night in mice (4), we hypothesized that the expression of these chaperones may be governed by circadian rhythm. In the experiments described below, we explored the connection between ER stress and circadian rhythm in fibroblasts actively secreting collagen.

## MATERIALS AND METHODS

### Cell isolation and culture

Tail tendon fibroblasts were released from the tail tendons of 6- to 8-wk-old period circadian regulator 2–luciferase (Per2::luc)–knock-in mice (kindly donated by Prof. Joseph Takahashi, University of Texas Southwestern Medical Center, Dallas, TX, USA) by 4 mg/ml bacterial collagenase type 4 (Worthington Biochemical, Lakewood, NJ, USA) in EDTA-free trypsin (2.5 g/L) as previously described in Yeung *et al*. (15). Cells were cultured in complete medium (DMEM: nutrient mixture F-12 containing 4500 mg/L glucose, l-glutamine, and sodium bicarbonate, supplemented with 10% fetal calf serum) at 37°C, in 5% CO_2_. Immortalized lines were generated by retroviral expression of murine TERT (16) [immortalized tail tendon fibroblast (iTTF)]; similarly, to generate BiP-overexpressing cells, we utilized pCMMP-BiP-IRES-mRFP, a gift from Bill Sugden (Addgene plasmid 36975) (17), using methods previously described in Pickard *et al*. (18). BiP-overexpressing cells were flow-sorted based on monomer red fluorescent (mRFP) protein expression. Stable cell lines expressing mutant thyroglobulin were generated by transfecting pcDNA3-neo-ColII-Tg-COG into iTTFs using Fugene 6 (Promega, Madison, WI, USA). Forty-eight hours after transfection, cells were maintained in 200 µg/ml neomycin for 2 wk. Prior to analysis of luminescence, neomycin was removed from culture medium, and control cells were transfected with pEGFP-N1 (Clontech Laboratories, Mountain View, CA, USA) and selected as previously mentioned.

### Luminometry

LumiCycle apparatus (Actimetrics, Wilmette, IL, USA) was used for real-time quantitative bioluminescence recording. Tail tendons, 10 mg, were placed in 30-mm cell culture inserts (0.4 µm pore size; MilliporeSigma, Burlington, MA, USA) inside 35-mm dishes in recording medium [DMEM without phenol-red (D2902; MilliporeSigma), supplemented with 4 g/L glucose, 5% fetal calf serum, HEPES, sodium bicarbonate, and 0.1 mM luciferin]. iTTFs were seeded 24 h, as previously described, prior to drug treatments. Dexamethasone (100 nM) was added for 30 min to synchronize the circadian rhythms before plating in recording medium. Baseline subtraction was carried out using a 24-h moving mean. Amplitude was calculated as peak-trough difference in bioluminescence of the first and second peak, as indicated, using baseline-subtracted data.

### Western blot

Proteins were extracted using urea buffer and analyzed by Western blotting as previously described in Pickard *et al*. (19). Primary antibodies used were mouse mAb to BiP (1:1000; sc-376768; Santa Cruz Biotechnology, Dallas, TX, USA), rabbit pAb to collagen-I (1:500; OARA02579; Gentaur, Kampenhout, Belgium), mouse mAb to glyceraldehyde-3-phosphate dehydrogenase (GAPDH; 1:10,000; clone GAPDH-71.1; MilliporeSigma), mouse mAb to vinculin (1:800; V9131; MilliporeSigma), and mouse mAb to β-actin (1:2000; sc-8432; Santa Cruz Biotechnology).

### Dose response curves

The appropriate dosages of thapsigargin and tunicamycin for *in vitro* treatments were determined in triplicate using the AlamarBlue assay (Thermo Fisher Scientific, Waltham, MA, USA) in 96-well format (20).

### Real-time PCR

For quantitative PCR analysis, primers designed using Assay Design Center (Roche, Basel, Switzerland), the sequences used are indicated in Supplemental Table S1. Reactions were run using Power-Up Sybr Master Mix (Thermo Fisher Scientific) with cDNA generated using a TaqMan Reverse Transcription Kit (Thermo Fisher Scientific). RNA (0.5 µg) extracted using Trizol (Thermo Fisher Scientific) was used to generate cDNA, which was then diluted 20-fold for PCR. PCR reactions were run on the CFX96 Real-Time System (Bio-Rad, Hercules, CA, USA). Target transcript expression was normalized to the geometric mean of ribosomal protein lateral stalk subunit P0 (RPLP0), *Gapdh*, and actin β.

### Immunofluorescence and in-cell Western blot

For immunofluorescence analysis cells were fixed with 4% paraformaldehyde and permeabilized with 0.5% Triton-X–PBS. Collagen-I was detected using a rabbit pAb (1:200; OARA02579; Gentaur), and ER was identified using a mouse mAb against protein disulfide isomerase (PDI) (1:100; ab190883; Abcam, Cambridge, MA, USA). Images were collected on a Leica TCS SP5 Acousto-Optical Beam Splitter inverted confocal using a ×60/0.50 Plan Fluotar objective and 3× confocal zoom (Leica Microsystems, Buffalo Grove, IL, USA). The confocal settings were as follows: pinhole 1 Airy unit, scan speed 1000 Hz unidirectional, format 1024 × 1024. Images were collected using photomultiplier tube detectors with the following detection mirror settings: FITC 494–530 nm; cyanine 5 (Cy5) 640–690 nm using the 488 nm (20%), 594 nm (100%), and 633 nm (100%) laser lines, respectively. To eliminate cross-talk between channels, the images were collected sequentially. When acquiring 3-dimensional optical stacks, the confocal software was used to determine the optimal number of *z* sections. Only the maximum intensity projections of these 3-dimensional stacks are shown in the results. For imaging nonhelical collagen, cells were permeabilized as above and stained with anticollagen-I (1:200, OARA02579; Gentaur) and 20 µM 5-carboxyfluorescein–conjugated collagen hybridizing peptide (3Helix, Salt Lake City, UT, USA). Cells were stained for 2 h at 4°C. Images were collected on a Zeiss Examiner A1 upright microscope using a ×63/1.4 N-Achroplan objective (Carl Zeiss, Oberkochen, Germany) and captured using a Coolsnap ES camera (Photometrics, Huntington Beach, CA, USA) through Metavue v.7.8.0.0 software (Molecular Devices, Sunnyvale, CA, USA). Specific band pass filter sets for DAPI, FITC, and cyanine 3 (Cy3) were used to prevent bleed-through from 1 channel to the next. For in-cell Western blots, iTTFs were grown in 96-well plates in the presence of 200 µM ascorbic acid; 24 h after plating, cells were treated with tunicamycin and thapsigargin as indicated before removing treatment and adding full medium supplemented with ascorbic acid and dexamethasone. After 72 h, cells were fixed with 4% paraformaldehyde. To assess extracellular collagen, cells were directly stained with an anti-collagen antibody (1:1000; OARA02579; Gentaur) overnight, which was detected using donkey anti-rabbit 800 antibody (1:10,000, 5151s; Cell Signaling Technology, Danvers, MA, USA); each well was counterstained with the cell-permeating dye deep red anthraquinone 5 (DRAQ5). The plate was scanned using the Odyssey CLx (Li-Cor Biosciences, Lincoln, NE, USA). The collagen signal was quantified following normalization to deep red anthraquinone 5. To assess intracellular collagen, the plates were permeabilized as for immunofluorescence studies before being stained and quantified in the same manner as extracellular collagen.

### Statistics

For assessment of data fit to circadian rhythms, microarray data were analyzed using the MetaCycle package for R (*https://CRAN.R-project.org/package=MetaCycle*), which combines 3 approaches (JTK_Cycle, Lomb-Scargle, and Arser) to decide on rhythmicity. Protein data were assessed using CircWaveBatch v.3.3 (*https://www.euclock.org/results/item/circ-wave-batch.html*). Where indicated, paired Student’s *t* tests were performed using triplicate analysis from at least 2 independent experiments. A value of *P* < 0.05 was deemed significant.

## RESULTS

### BiP is circadian clock rhythmic

**Figure 1:**
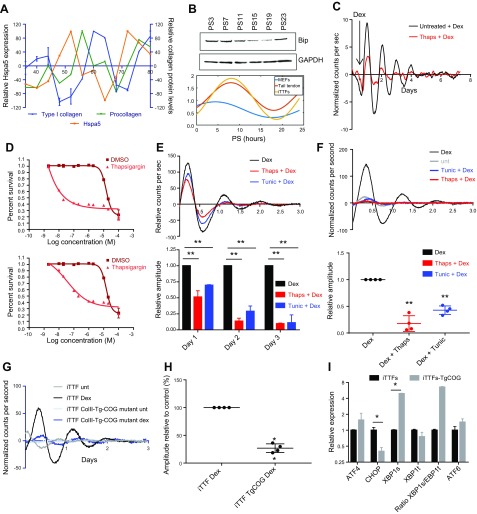
ER stress dampens circadian rhythm. *A*) Hspa5 (BiP or GRP78) transcript levels fluctuate over the course of 2 d in mouse tail tendons as assessed by microarray; *n* = 2 animals/time point. BiP levels rise prior to the increased synthesis of collagen, indicated by procollagen-specific peptides; *n* = 4 animals/time point. *X* axis represents hours in dark/dark cycle, as previously described in Yeung *et al*. (34). *B*) BiP levels as assessed by Western blot in MEFs over the course of 24 h postsynchronization (PS) with 50% horse serum. Lower traces show the fluctuations in BiP protein levels, assessed by densitometry and normalized to GAPDH, in tail tendons, iTTFs, and MEFs as assessed by CircWave (see Supplemental Fig. S1). *C*) The effects of ER stress induction on circadian rhythm in *ex vivo* tail tendons from Per2::luc mice. Tendons were treated with 10 nM thapsigargin in recording medium and then treated with dexamethasone to entrain the circadian rhythm. *D*) Cell viability after 72 h in response to the indicated doses of thapsigargin (upper) and tunicamycin (lower) as assessed by AlamarBlue (*n* = 3); error bars represent sd. *E*) Representative luminescence traces (upper) following 1 h treatment of 10 nM thapsigargin or 100 ng/ml tunicamycin, which dampens the induction of Per2::luc in response to dexamethasone; relative amplitude over 3 d is shown (lower); *n* = 3 biologic replicates. *F*) Thapsigargin and tunicamycin are removed from Per2::luc cells after 5 h treatment, then synchronized with dexamethasone and then compared to untreated cells. Relative amplitudes in the first 24 h are shown (lower chart, *n* = 4 biologic replicates). *G*) iTTFs expressing congenital goiter–mutated thyroglobulin (ColII-Tg-COG mutant) have reduced inherent and dexamethasone-induced rhythms; *n* = 2 independently generated cell lines; *n* = 2 biologic replicates. *H*) Quantification of amplitudes of Per2::luc traces. *I*) Levels of spliced XBP1 (XBP1s) and total Xbp1 (XBP1t) indicate that the IRE1-XBP1 arm of the UPR has been activated in ColII-Tg-COG cells; *n* = 3; error bars represent sd. Dex, dexamethasone; Thaps, thapsigargin; Tunic, tunicamycin; unt, untreated. *P < 0.05, **P < 0.01 (paired Student’s *t* test).

BiP is known to interact with collagen in the secretory pathway (21) and is proposed to aid the folding of collagen. As a first experiment, we showed that levels of BiP are rhythmic with a 24-h period (Metacycle Benjamini–Hochberg *q*-value 0.003) and its peak of expression precedes the peak of expression of pro–collagen-I (the precursor of collagen) protein (**Fig. 1*A***). The circadian nature of BiP protein expression was also observed in mouse embryonic fibroblasts (MEFs), isolated tail tendon fibroblasts, and tail tendon tissue with peak and minimal expression (Fig. 1*B* and Supplemental Fig. S1). The fluctuations in BiP levels during the day support recent observations demonstrating that BiP is a short-lived protein that is turned over in 2–3 h (22). Examination of chromatin immunoprecipitation sequencing data sets (23) showed that the *Hspa5*/BiP promoter is differentially bound by the core clock component cryptochrome protein 2 (Cry2) during the course of 24 h, with RNA polymerase II being recruited at times corresponding to elevated BiP transcription (Supplemental Fig. S1) This result was the first indication that the circadian clock had a role in preemptively averting ER stress by up-regulating BiP in anticipation of a surge in collagen synthesis.

### The presence of misfolded protein ablates circadian rhythm

To learn more about the effects of ER stress on the circadian rhythm, we treated the tail tendons of *ex vivo* Per2::luc clock reporter mice (2) with thapsigargin. Thapsigargin inhibits the sarcoplasmic or ER Ca-ATPase family, which leads to a reduction of ER calcium levels and so induces protein misfolding and ER stress (24). When cells were subjected to dexamethasone to synchronize the circadian clock, the amplitude of rhythm was markedly reduced after thapsigargin treatment (Fig. 1*C*). Similarly, when iTTFs from Per2::luc mice were pretreated for 1 h with thapsigargin or tunicamycin (an inhibitor of *N*-linked glycosylation, which normally aids protein folding) at doses that do not impact on cell growth (Fig. 1*D*), the amplitude of Per2::luc rhythm was dampened (Fig. 1*E*).

This connection between ER stress and circadian rhythm was further exemplified in cells that were in the process of resolving ER stress prior to dexamethasone treatment. We used a 5-h treatment with thapsigargin or tunicamycin to accumulate misfolded proteins in the ER and induce all arms of the UPR (Supplemental Fig. S2) prior to synchronization of the circadian rhythm. This treatment regime eliminated the synchronization effect of dexamethasone and forskolin (Fig. 1*F* and Supplemental Fig. S3), which suggested that cells undergoing ER stress do not have a functioning circadian clock. Similarly, in cultures with a preestablished rhythm, treatment with thapsigargin flattened Per2::luc fluctuations (Supplemental Fig. S3).

To establish if misfolded proteins instigate the dampening of circadian rhythms, we used a mutated thyroglobulin that misfolds in the ER, thereby causing ER stress (25). When this mutated thyroglobulin was stably expressed in Per2::luc cells, there was dramatic dampening of circadian rhythm (Fig. 1*G, H*) and induction of ER stress, particularly increasing the splicing of X-box binding protein 1 (XBP1) (Fig. 1*I*). Thus, the fact that circadian rhythm is lost in fibroblasts expressing misfolded thyroglobulin shows that the loss of rhythm is the direct consequence of accumulated misfolded protein.

### Suppression of the secretory pathway blocks circadian rhythm by induction of ER stress

**Figure 2:**
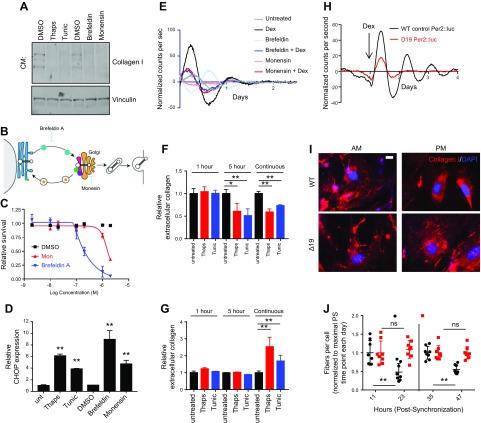
Impact of ER stress on collagen production. *A*) Western blot analysis on conditioned medium (CM) collected from iTTFs after 48 h of treatment with thapsigargin, tunicamycin, brefeldin, and monensin suggests that secreted collagen-I is suppressed in treated cells (*n* = 2). *B*) Diagram of the secretory pathway marking the position of action for the inhibitors brefeldin A and monensin. *C*) The effects of brefeldin A and monensin, at various concentrations, on the survival of fibroblasts following 72 h treatment. *n* = 3; error bars represent sd. *D*) The effects of brefeldin A and monensin, at various concentrations, on the survival of fibroblasts following 72-h treatment. *E*) Levels of CHOP/DDIT3 indicate that the unfolded protein response has been activated following 5-h treatment with brefeldin A and monensin, similar to the effects of thapsigargin and tunicamycin. *F*, *G*) Using in-cell Western blot, the effects of thapsigargin and tunicamycin on the production of extracellular collagen (*F*) and intracellular collagen (*G*) are shown, with the effects of pretreatment with thapsigargin or tunicamycin for different times; error bars represent sd of triplicate measurements. *H*) Per2::luc signals in lung fibroblasts isolated from wild-type control and ClockΔ19 mice. Cells were recorded for 24 h before treatment with 100 nM dexamethasone at the indicated time. Representative traces are shown; *n* = 3. *I*) Representative images of collagen fibers formed by WT and ClockΔ19 fibroblasts 12 h apart (am and pm, d 2) after synchronization with dexamethasone. Scale bar, 10 μm. *J*) Scores of the number of collagen fibers per cell were assessed over 2 d; peak numbers of collagen fibers are observed 11 h after the synchronization event; data on each day are normalized to this time point: 11 h postsynchronization or 35 h postsynchronization (11 h postsynchronization + 24 h). On average, 2000 cells per time point were scored. Δ19, ClockΔ19; Dex, dexamethasone; Mon, monensin; n.s., not significant; Thaps, thapsigargin; Tunic, tunicamycin; unt, untreated; WT, wild type. *P < 0.05; **P < 0.01 (paired Student’s *t* test).

The effect of ER stress induction on collagen secretion was examined by Western blot analysis of the conditioned medium of thapsigargin- or tunicamycin-treated cells using an anti–collagen-I antibody. Over 48 h, both thapsigargin and tunicamycin abolished secretion of collagen-I (**Fig. 2*A***). To examine if blockade of the secretory pathway had a similar effect on the secretion of collagen, cells were treated with brefeldin A and monensin (Fig. 2*B, C*). These treatments suppressed collagen secretion and induced ER stress, as shown by induction of CCAAT-enhancer binding protein–homologous protein (CHOP) transcription after 5 h treatment (Fig. 2*D*). These effects were comparable to those of thapsigargin and tunicamycin, and likewise, both brefeldin A and monensin dampened circadian fluctuations in Per2::luc cells (Fig. 2*E*).

Having demonstrated that short-term treatment with thapsigargin or tunicamycin dampens circadian rhythm, we sought to utilize this to assess the importance of circadian rhythm on the secretion and assembly of collagen fibers. First, we showed that after treatment with 100 nM dexamethasone, there is enhanced fibril assembly in iTTFs (Supplemental Fig. S4); these short-term effects of dexamethasone have previously been demonstrated in refs. 26 and 27 in fibroblasts. We used a modified in-cell Western blot approach to demonstrate the effects of thapsigargin and tunicamycin on collagen secretion (Fig. 2*F, G*). As expected, continuous treatment of fibroblasts with ER stress inducers reduced the assembly of extracellular collagen (Fig. 2*F*), which was accompanied by accumulation of intracellular collagen (Fig. 2*G*), in line with reduced secretion of collagen into the medium (Fig. 2*A*). Having validated the in-cell Western blot, we then induced ER stress using a 5-h pulse of thapsigargin or tunicamycin prior to dexamethasone treatment. The results showed that disruption of rhythm reduced the accumulation of extracellular collagen after 72 h, implicating circadian rhythms in coordinating collagen secretion and assembly. Importantly, the intracellular levels of collagen were unaffected by this treatment regime, indicating that collagen had not been held in the ER. A 1-h treatment did not affect either extracellular or intracellular collagen. We have evaluated collagen fiber assembly in fibroblasts isolated from the circadian locomotor output cycles Kaput Δ19 (ClockΔ19) *N*-ethyl-*N*-nitrosoure (ENU) mutation mouse, a mutation which is a deletion in exon 19 of the Clock gene resulting in diminished circadian activity (28). These fibroblasts lack a sustained Per2::luc rhythm in response to dexamethasone treatment (Fig. 2*H*). In cultures of ClockΔ19 fibroblasts, there were increased numbers of collagen fibers compared with wild-type fibroblasts (Fig. 2*I, J*) and reduced ER stress–related transcripts (Supplemental Fig. S5), which supports recent observations that showed increased collagen deposition in the tendons of ClockΔ19 mice (4). The deposition of collagen fibers by fibroblasts fluctuates during the day (4). These time-dependent changes in collagen are not observed in cultures of ClockΔ19 fibroblasts (Fig. 2*I*), suggesting that circadian rhythm is required for normal collagen fiber homeostasis. Together these results suggest that ER stress can regulate circadian rhythm and that circadian rhythm affects collagen fiber homeostasis, but that collagen secretion *per se* does not require an intact circadian rhythm.

### BiP retains collagen in the ER but maintains circadian rhythm

**Figure 3:**
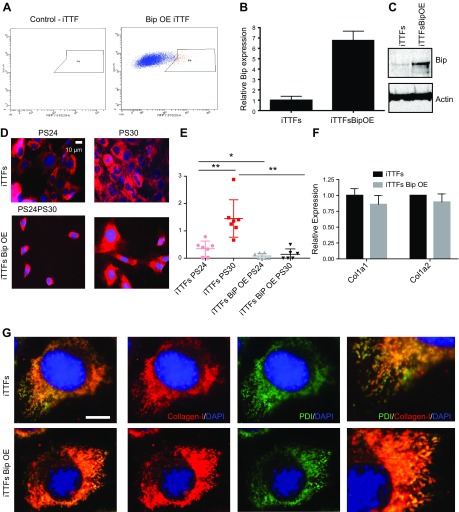
BiP retains collagen in the ER. *A*) iTTFs were transduced with pCMMP-BiP-IRES-mRFP; red fluorescent protein (RFP)–positive cells were sorted to form a BiP-overexpressing cell line (iTTF + BiP). *B*, *C*) Levels of BiP mRNA (*B*) and protein (*C*) were assessed in sorted populations. *D*) Immunofluorescence detection of collagen fibers in control and BiP-overexpressing iTTFs; cells were synchronized with dexamethasone and collected at different time points. Scale bar, 10 μm. *E*) Scores of the numbers of collagen fibers counted per cell at the indicated times; *n* = 2 biologic repeats, with 3–4 regions scored per sample; ∼200 cells per region were scored. *P < 0.05, **P < 0.01 (paired Student’s *t* test). *F*) The effects of BiP overexpression on the transcript levels of collagen-I (Col1a1 and Col1a2); *n* = 2 biologic repeats. *G*) Colocalization of collagen-I and the ER marker PDI in control and BiP-overexpressing iTTFs. OE, overexpressing; PS24 and PS30, 24 and 30 h postsynchronization of the circadian clock by dexamethasone. Scale bar, 10 μm.

**Figure 4:**
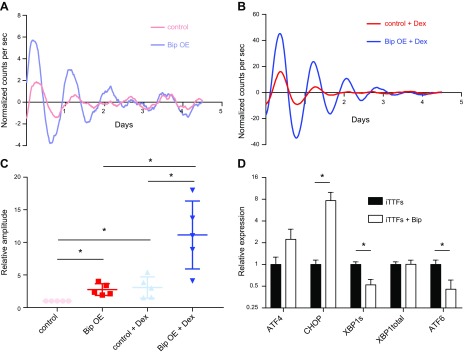
BiP overexpression strengthens circadian rhythm. *A*, *B*) Representative traces of control and BiP-overexpressing iTTFs; BiP-expressing cells have a more robust circadian rhythm in unsynchronized populations (*A*) and following dexamethasone-induced synchronization (*B*). *C*) Amplitude of Per2::luc signals from control and BiP-overexpressing iTTFs, unsynchronized and synchronized by dexamethasone, in the first 48 h, quantified from *n* = 3 biologic replicates; error bars represent sd. *D*) The activation of the UPR in BiP-overexpressing cells was assessed by monitoring the expression of ATF4/CHOP, Xbp1 splicing (XBP1s), and ATF6 transcripts; *n* = 2, each analyzed in triplicate, error bars represent sd. Dex, dexamethasone; OE, overexpressing. *P < 0.05 (paired Student’s *t* test).

Given the circadian rhythmicity of BiP protein levels, which peaks ahead of collagen-I (see Fig. 1*A*), we examined how BiP overexpression affects collagen-I secretion and the circadian rhythm. BiP was overexpressed in iTTFs using retroviral transduction (**Fig. 3*A***), which was confirmed by real-time PCR and Western blotting (Fig. 3*B, C*). BiP overexpression led to inhibition of collagen fiber assembly at 2 time points following synchronization (Fig. 3*D, E*). Both dexamethasone and serum shock induced a significant increase in collagen fibers in synchronized cultures (Supplemental Fig. S4), implying that synchronization can aid the assembly of collagen fibers. Forskolin had the opposite effect (Supplemental Fig. S4); although forskolin can synchronize the circadian clock, it also increases cAMP levels, which has previously been demonstrated to inhibit collagen trafficking (29) and TGF-β induced collagen production (30). Based on this, we have observed that addition of the cell-permeable cAMP analog can suppress collagen synthesis in the presence of dexamethasone (Supplemental Fig. S4). In control cells, fiber assembly was observed at times after BiP levels had peaked (see Fig. 1*B*). Overexpression of BiP did not alter the transcription of either Col1a1 or Col1a2 (Fig. 3*F*), suggesting that the reduced assembly of collagen fibers is a result of altered collagen transport through the secretory pathway. Immunofluorescence detection of intracellular collagen in BiP-overexpressing cells showed that the majority of the collagen colocalizes with the ER marker PDI, whereas in control cells, regions of the ER are clear from collagen. Thus, collagen is retained in the ER when BiP is overexpressed (Fig. 3*G*). The use of collagen-hybridizing peptide (31) indicated that the collagen held in the ER of BiP-overexpressing cells is largely unfolded (Supplemental Fig. S6). Therefore, we examined the impact of BiP overexpression on circadian rhythm. To our surprise, the cells have a stronger inherent rhythm (**Fig. 4*A***) and increased amplitude of Per2::luc signals following dexamethasone treatment (Fig. 4*B, C*). In a recent genome-wide small interfering RNA screen (32), knockdown of BiP was shown to suppress circadian rhythm (Supplemental Fig. S7). BiP is well known to suppress the activation of all 3 arms of the UPR; therefore, this result implies that the activation of the UPR arms is responsible for the dampening of circadian rhythms in cells undergoing ER stress. Real-time PCR analysis showed reduced levels of the ATF6 and IRE1-XBP1 arms of the UPR in BiP-overexpressing cells; however, there is enhanced activation of the ATF4 arm, as shown by elevated CHOP expression (Fig. 4*D*).

### Protein folding in the ER provides a checkpoint at which circadian rhythm can be rapidly controlled

**Figure 5:**
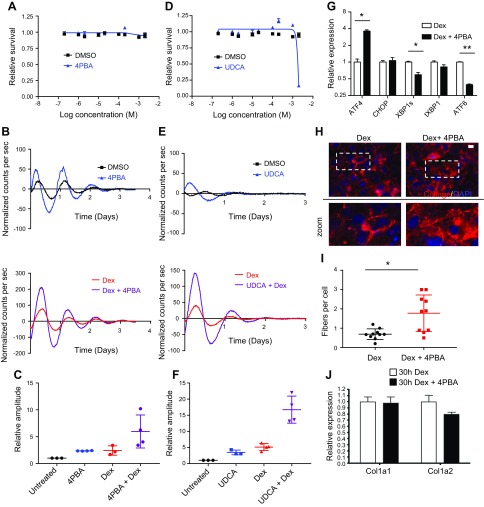
Chemical chaperones strengthen circadian rhythm and enhance collagen secretion. *A*) Effects of 4PBA treatment on the survival of iTTFs as assessed by AlamarBlue; triplicate analysis, error bars represent sd. *B*) The effects of 100 µM 4PBA on circadian rhythm in unsynchronized populations (upper panel) and following dexamethasone treatment (lower panel); representative traces are shown. *C*) Amplitudes of Per2::luc signals following 4PBA treatment; *n* = 3–4 biologic replicates. *D*) Effects of UDCA treatment on the survival of iTTFs as assessed by AlamarBlue; triplicate analysis, error bars represent sd. *E*) The effects of 100 µM UDCA on circadian rhythm in unsynchronized populations (upper panel) and following dexamethasone (lower panel). *F*) Amplitudes of Per2::luc signals following UDCA treatment. *n* = 3–4 biologic replicates. *G*) Effects of 4PBA on the expression of components of the UPR [ATF4, CHOP, spliced XBP1 (XBP1s), total XBP1 (tXBP1), ATF6] after 72 h treatment; *n* = 2 each analyzed in triplicate; error bars represent sem. *H*, *I*) The effects of 4PBA treatment on the production of collagen fibers following 72-h treatment (*H*) and quantified (*I*); *n* = 2 with 5 regions scored in each sample; ∼200 cells/region were scored; error bars represent sd. Scale bar, 10 μm. *J*) The impact of 4PBA treatment on collagen-I transcripts (Col1a1 and Col1a2); *n* = 2 each analyzed in triplicate; error bars represent sd. Dex, dexamethasone. *P < 0.05, **P < 0.01 (paired Student’s *t* test).

The influence of unfolded proteins on circadian clock regulation was explored using clinically approved chemical chaperones, which aid the folding of proteins within the ER (33). 4-Phenylbutyric acid (4PBA) and ursodeoxycholic acid (UDCA) induced a visible rhythm in fibroblasts but also greatly increased the amplitude of Per2::luc signals following dexamethasone treatment (**Fig. 5*A–F***). These results resemble the effects observed with BiP overexpression and suggest that misfolded proteins are resident in the ER of cultured cells and, through promoting their folding, can rescue dampened circadian rhythms. The chaperone inhibitor 2-phenylethynesulfonamide had the opposite effect, reducing Per2::luc amplitudes, which was most likely due to the observed induction of the UPR (Supplemental Fig. S8). Assessment of the ER stress pathways following 4PBA treatment showed that there is reduced expression of both ATF6 and IRE1-XBP1 arms of the UPR, again mirroring the effects of BiP overexpression, and although ATF4 expression was enhanced, there was no elevated expression of CHOP (Fig. 5*G*). Treatment of tendon fibroblasts with 4PBA also led to enhanced secretion of collagen fibers (Fig. 5*H, I*) but without altering transcription of collagen-I (Fig. 5*J*). Taken together, these findings imply that protein folding in the ER is a rate-limiting step in collagen biosynthesis and provides a checkpoint for control of the circadian clock.

## DISCUSSION

The intrinsic circadian timing mechanism allows a cell to anticipate and adapt to daily rhythmic changes in physiologic demand. In this study we have shown that BiP is rhythmic in tendon and cultured fibroblasts and that the peak of BiP expression occurs just ahead of the peak of pro–collagen-I expression. We further showed that ER stress–inducing agents or expression of misfolded ER proteins weakens circadian rhythm and decreases collagen secretion. The use of chemical chaperones to aid protein folding shows that the resolution of ER stress can also enhance circadian rhythm and collagen deposition.

Yeung *et al.* (34) showed that tendon tissue (with a composition of 80–90% collagen) has an autonomous circadian clock and identified 745 rhythmic transcripts that had a 24-h oscillation. However, transcripts encoding collagens were not rhythmic. In a subsequent study, Yeung *et al.* (4) demonstrated that the protein levels of collagen-I oscillate with a 24-h period, suggesting that the circadian rhythmicity of collagen-I is generated post-transcriptionally. The ramp in procollagen concentration in the ER at night might be expected to lead to ER stress if the levels of nascent unfolded protein are allowed to escalate unchecked, because the levels of BiP are also circadian-regulated and peak a few hours ahead of the peak of procollagen. This suggests that the circadian clock prepares the cell for the surge in nascent procollagen chains being synthesized. We propose that after the surge of procollagen has passed, BiP levels reduce to avoid swamping the ER stress sensors and thereby reestablish homeostasis to the UPR sensing machinery. We have demonstrated here that induction of ER stress can dampen the circadian rhythm in normal fibroblasts, suggesting that this surge of BiP may influence how cells perceive external circadian cues. The fluctuations in BiP levels appear to play a protective role in maintaining circadian rhythms, as observed with BiP overexpression. This is presumably because of the saturation of all 3 sensors of the UPR. Thus, we propose that the circadian clock has a role in preemptively preventing ER stress in normal cells, by up-regulating BiP in anticipation of a surge in protein translation. However, overexpression of BiP led to retention of procollagen in the ER, which is similar to what has been observed for other proteins (35, 36), notably that the retained procollagen was in a nonhelical conformation concomitant with reduced assembly of collagen fibers. A circadian control of BiP levels suggests that a balance is struck to have sufficient BiP to allow saturation of the UPR sensors and a robust circadian rhythm, but not too much to inhibit folding of procollagen.

We have shown that BiP overexpression and chemical chaperone treatment lead to suppression of Xbp1 splicing and ATF6 transcription. This suggests that these arms of the UPR are integrated into a feedback control of the molecular clock during ER stress. In these experiments, the ATF4 arm is largely unaffected; ATF4-knockout MEFs have previously been shown (37) to have reduced amplitude of Per2::luc rhythms, suggesting this pathway is essential for the robust rhythms, which may explain why these pathways remain active while the other arms of the UPR are suppressed. Of note, we have shown that ER stress or misfolded proteins can dampen clock activation in normal fibroblasts, whereas in a recent study in cancer cells undergoing ER stress a 10 h shift in circadian rhythm was observed (14). This suggests that cancer cells may negate the suppressive role of the UPR on the clock in order to survive. It may be that in a normal cell undergoing ER stress, the uncoupling of circadian clock control might facilitate the decision to resolve stress or undergo apoptosis. It has been proposed that cancer cell survival is enhanced through suppression of Clock and the brain and muscle ARNT-like 1 protein (Bmal1) (14), and both ER stress and UPR activation are well documented in many cancers, implicating the accumulation of misfolded proteins in these cells (38). Similarly, ER stress is active in patients with idiopathic pulmonary fibrosis (39), liver fibrosis (40), and kidney fibrosis (41, 42). Given that activation of ER stress would normally lead to dampening of circadian rhythms and collagen synthesis, this suggests that cells within diseased tissues have circumvented this physiologic control in order to drive fibrosis. Establishing how this is achieved in diseased tissues will be the focus of future studies.

## Supplementary Material

This article includes supplemental data. Please visit *http://www.fasebj.org* to obtain this information.

## Abbreviations

4PBA: 4-phenylbutyric acid
ATF: activating transcription factor
BiP: binding immunoglobulin protein
COL1A1: collagen-I α1 chain
COL1A2: collagen-I α2 chain
CHOP: CCAAT-enhancer binding protein–homologous protein
collagen-I: type I collagen
ClockΔ19: circadian locomotor output cycles Kaput Δ19
ER: endoplasmic reticulum
GAPDH: glyceraldehyde-3-phosphate dehydrogenase
Grp: glucose-regulated protein
Hsp: heat shock protein
IRE1: inositol-requiring enzyme 1
iTTF: immortalized tail tendon fibroblast
MEF: mouse embryonic fibroblast
PDI: protein disulfide isomerase
Per2::luc: period circadian regulator 2–luciferase
UDCA: ursodeoxycholic acid
UPR: unfolded protein response
XBP1: X-box binding protein 1
