# Supplementary material for: Preservation of circadian rhythms by the protein folding chaperone, BiP
Source: FASEB J. 2019 Mar 19;33(6):7479–89. doi: 10.1096/fj.201802366RR (PMC6529331; doi:10.1096/fj.201802366RR)
Supplement: Supplementary file 1 [file fj.201802366RR.sf1.pptx]

## Slide 1
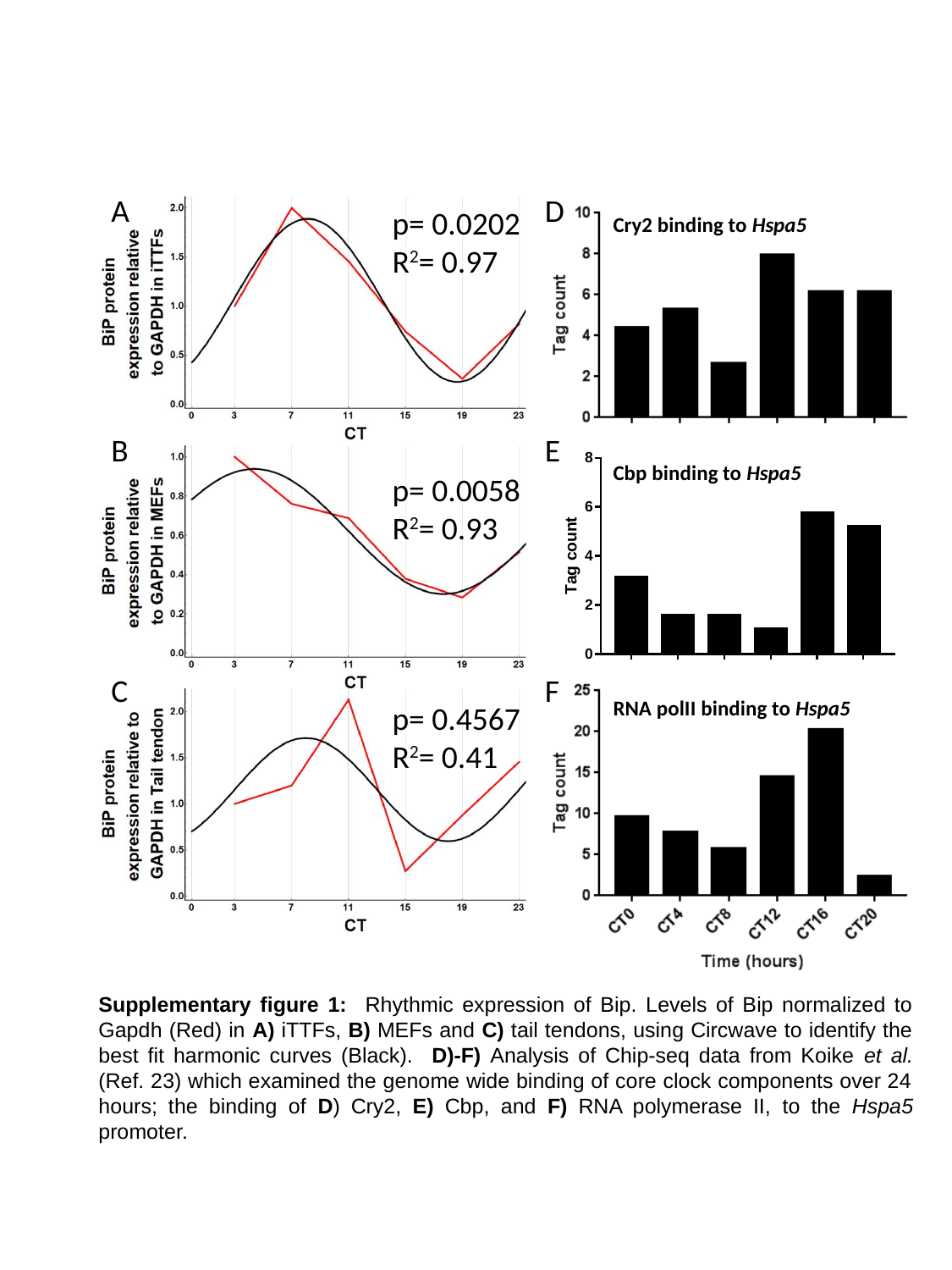

A
D
p= 0.0202
R2= 0.97
p= 0.0058
R2= 0.93
p= 0.4567
R2= 0.41
Cry2 binding to Hspa5
B
E
Cbp binding to Hspa5
C
F
RNA polII binding to Hspa5
Supplementary figure 1: Rhythmic expression of Bip. Levels of Bip normalized to Gapdh (Red) in A) iTTFs, B) MEFs and C) tail tendons, using Circwave to identify the best fit harmonic curves (Black). D)-F) Analysis of Chip-seq data from Koike et al. (Ref. 23) which examined the genome wide binding of core clock components over 24 hours; the binding of D) Cry2, E) Cbp, and F) RNA polymerase II, to the Hspa5 promoter.

## Slide 2
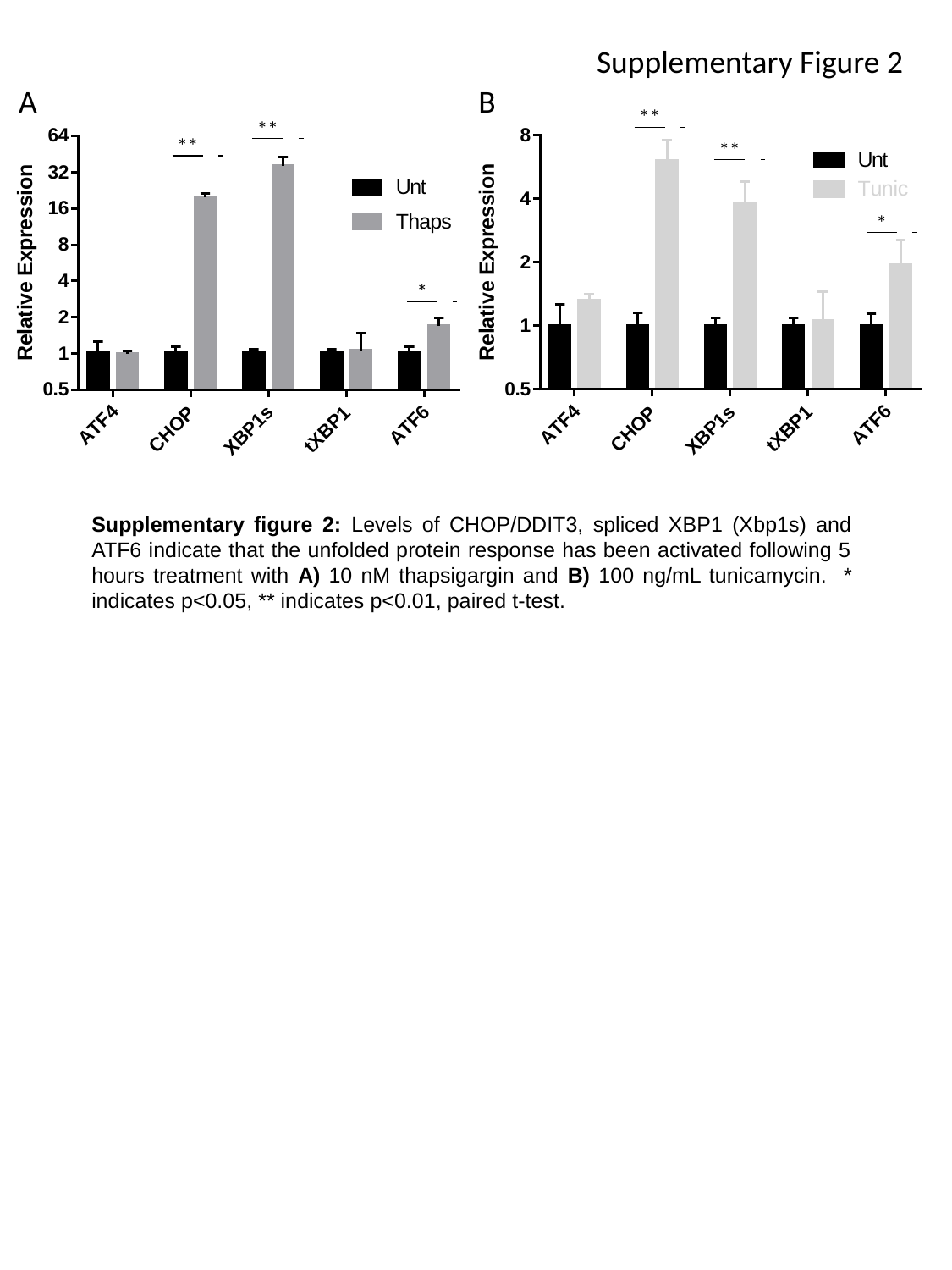

Supplementary Figure 2
A
B
 ** *
 ** *
 ** *
 ** *
 * *
 * *
Supplementary figure 2: Levels of CHOP/DDIT3, spliced XBP1 (Xbp1s) and ATF6 indicate that the unfolded protein response has been activated following 5 hours treatment with A) 10 nM thapsigargin and B) 100 ng/mL tunicamycin. * indicates p<0.05, ** indicates p<0.01, paired t-test.

## Slide 3
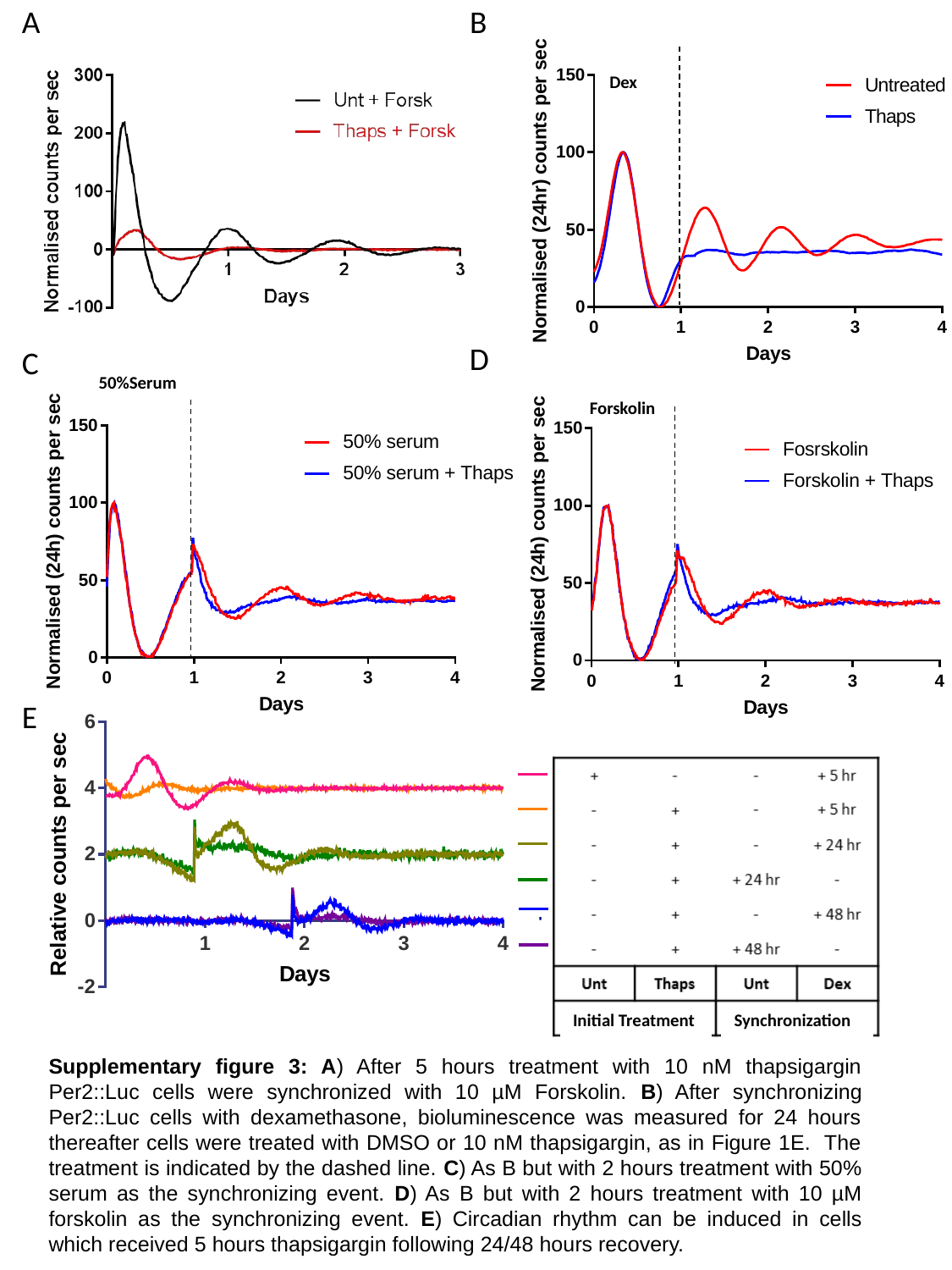

A
B
Dex
D
C
50%Serum
Forskolin
E
Initial Treatment
Synchronization
Supplementary figure 3: A) After 5 hours treatment with 10 nM thapsigargin Per2::Luc cells were synchronized with 10 µM Forskolin. B) After synchronizing Per2::Luc cells with dexamethasone, bioluminescence was measured for 24 hours thereafter cells were treated with DMSO or 10 nM thapsigargin, as in Figure 1E. The treatment is indicated by the dashed line. C) As B but with 2 hours treatment with 50% serum as the synchronizing event. D) As B but with 2 hours treatment with 10 µM forskolin as the synchronizing event. E) Circadian rhythm can be induced in cells which received 5 hours thapsigargin following 24/48 hours recovery.

## Slide 4
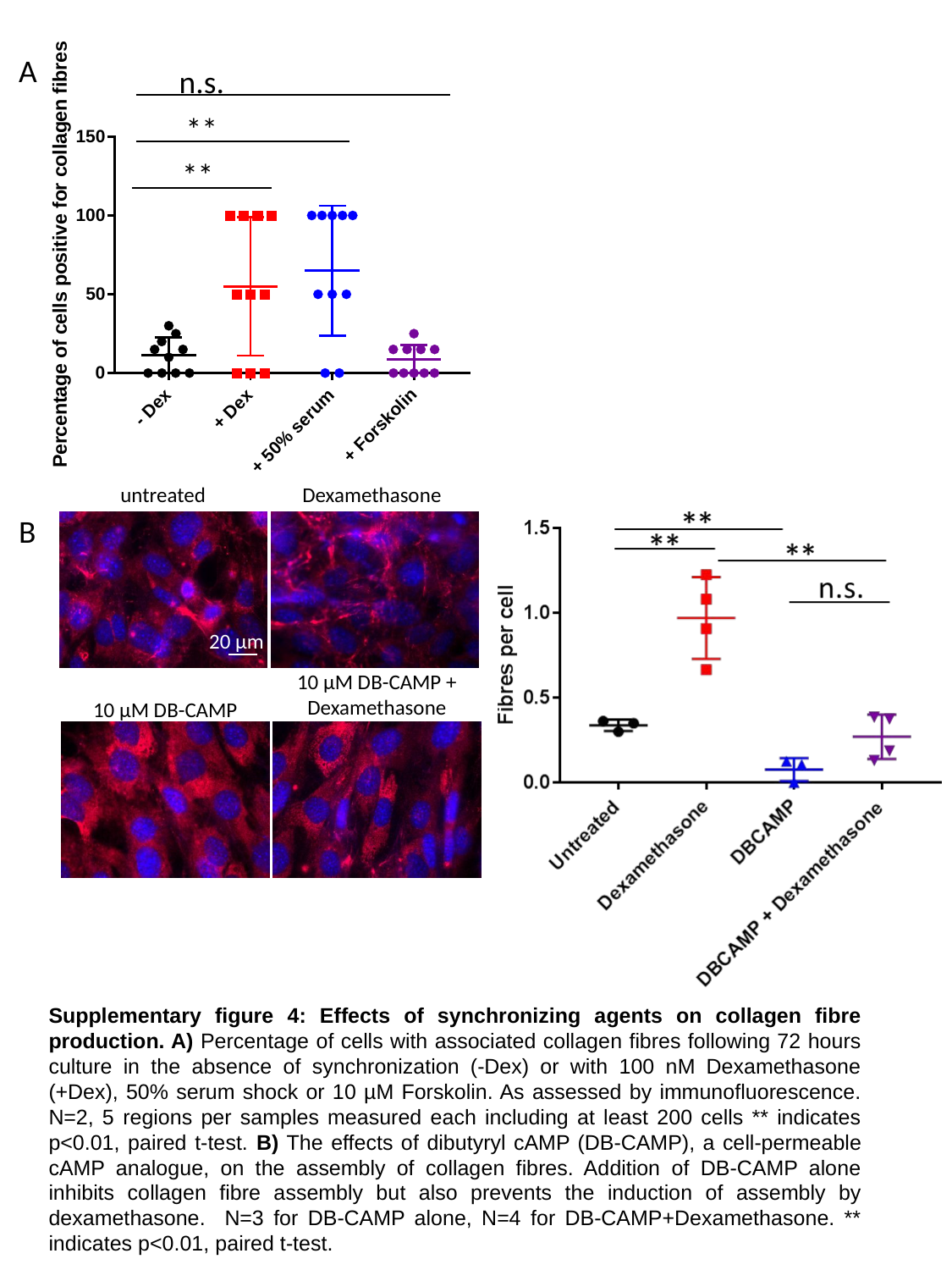

n.s.
**
**
A
untreated
Dexamethasone
20 µm
10 µM DB-CAMP + Dexamethasone
10 µM DB-CAMP
B
Supplementary figure 4: Effects of synchronizing agents on collagen fibre production. A) Percentage of cells with associated collagen fibres following 72 hours culture in the absence of synchronization (-Dex) or with 100 nM Dexamethasone (+Dex), 50% serum shock or 10 µM Forskolin. As assessed by immunofluorescence. N=2, 5 regions per samples measured each including at least 200 cells ** indicates p<0.01, paired t-test. B) The effects of dibutyryl cAMP (DB-CAMP), a cell-permeable cAMP analogue, on the assembly of collagen fibres. Addition of DB-CAMP alone inhibits collagen fibre assembly but also prevents the induction of assembly by dexamethasone. N=3 for DB-CAMP alone, N=4 for DB-CAMP+Dexamethasone. ** indicates p<0.01, paired t-test.

## Slide 5
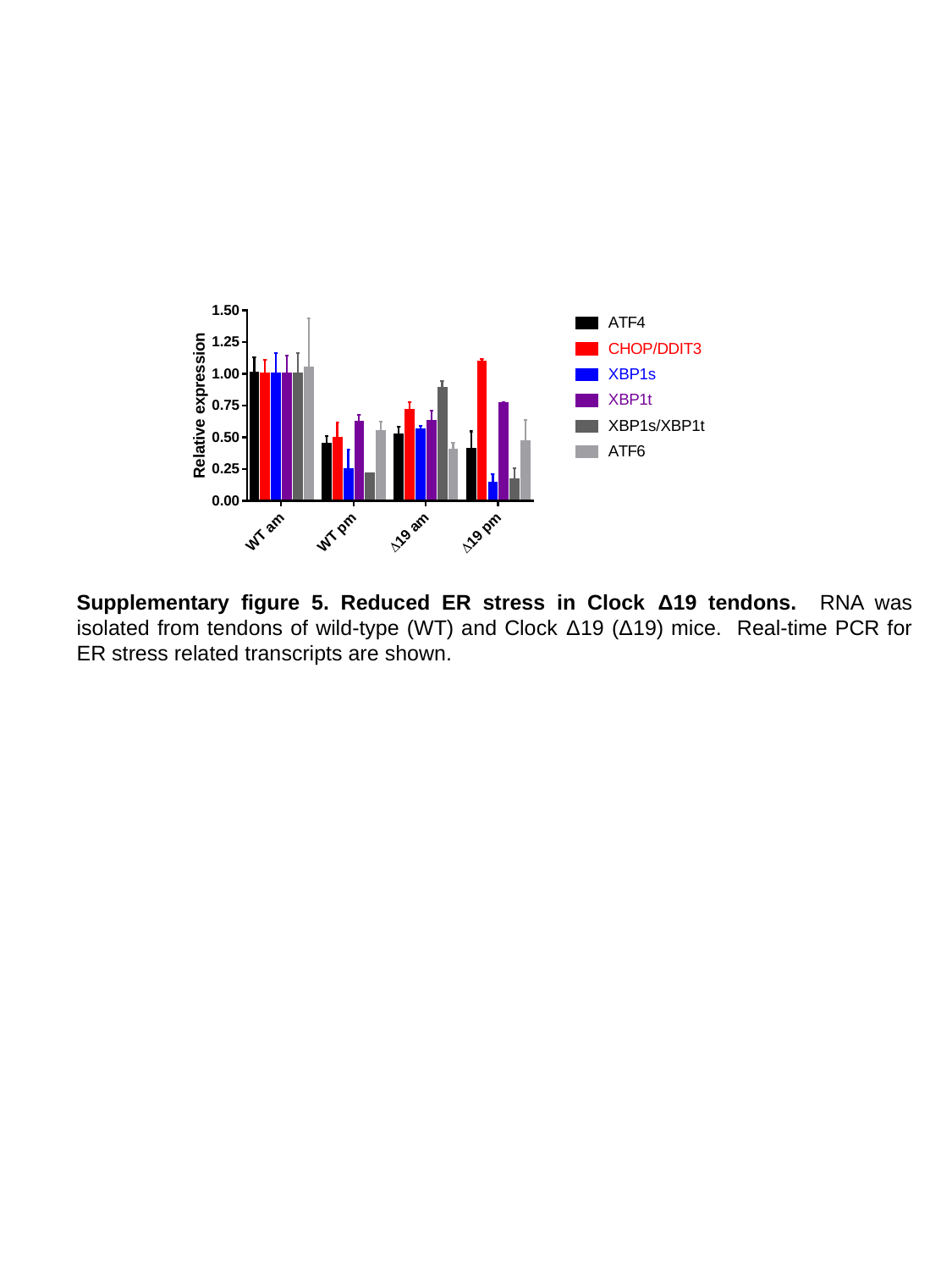

Supplementary figure 5. Reduced ER stress in Clock Δ19 tendons. RNA was isolated from tendons of wild-type (WT) and Clock Δ19 (Δ19) mice. Real-time PCR for ER stress related transcripts are shown.

## Slide 6
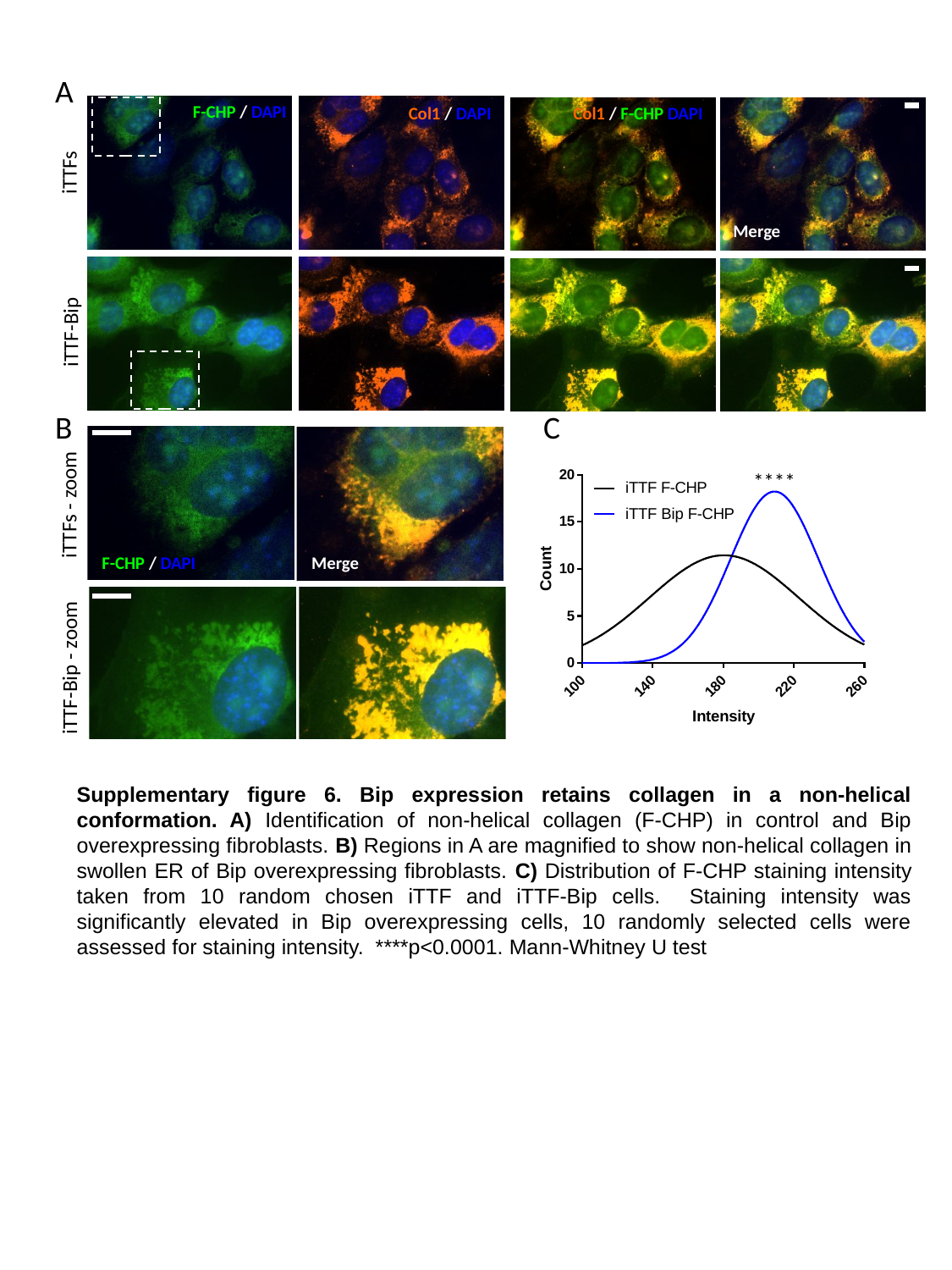

A
F-CHP / DAPI
Col1 / DAPI
Col1 / F-CHP DAPI
iTTFs
Merge
iTTF-Bip
B
C
****
iTTFs - zoom
F-CHP / DAPI
Merge
iTTF-Bip - zoom
Supplementary figure 6. Bip expression retains collagen in a non-helical conformation. A) Identification of non-helical collagen (F-CHP) in control and Bip overexpressing fibroblasts. B) Regions in A are magnified to show non-helical collagen in swollen ER of Bip overexpressing fibroblasts. C) Distribution of F-CHP staining intensity taken from 10 random chosen iTTF and iTTF-Bip cells. Staining intensity was significantly elevated in Bip overexpressing cells, 10 randomly selected cells were assessed for staining intensity. ****p<0.0001. Mann-Whitney U test

## Slide 7
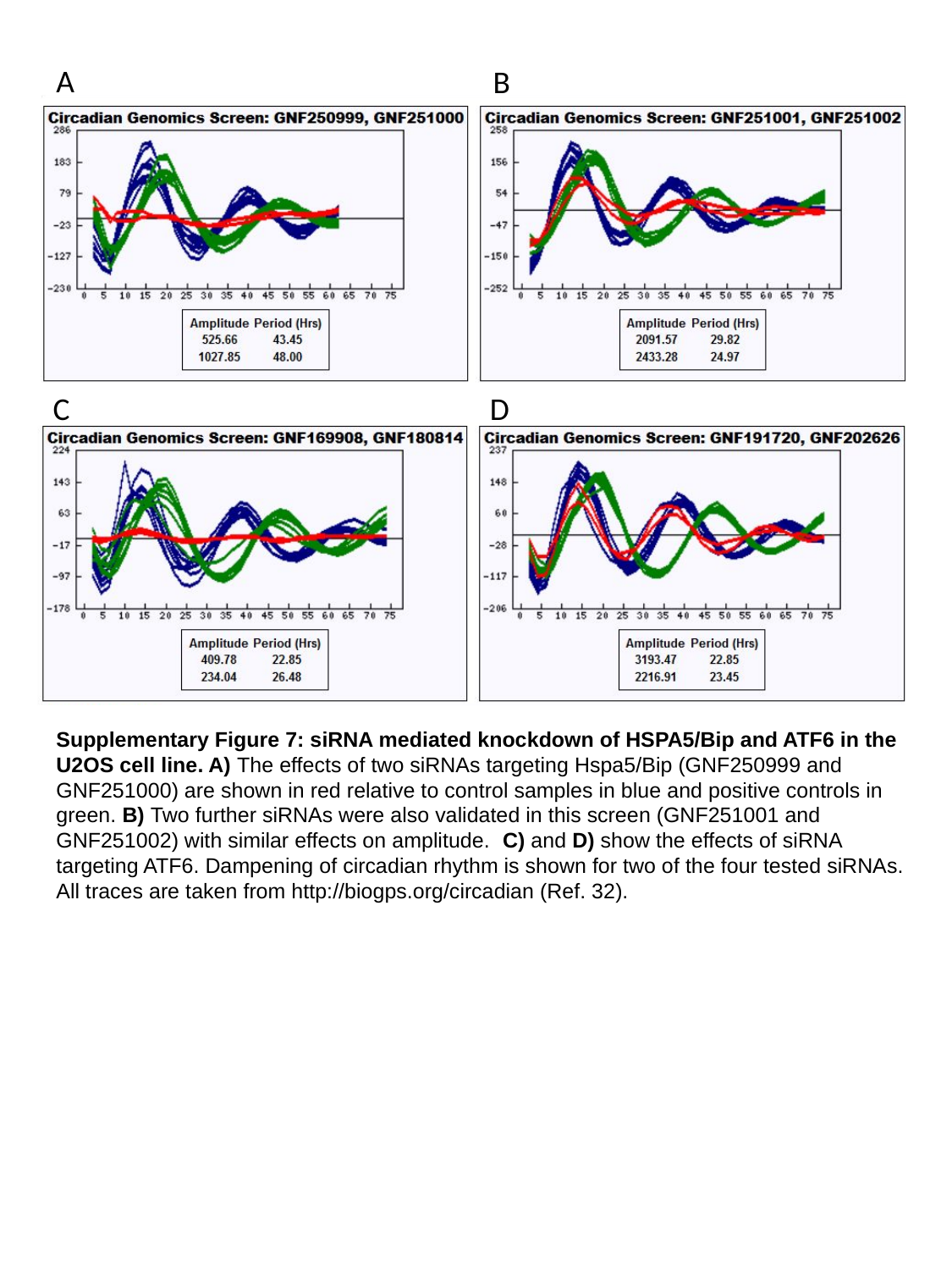

A
B
C
D
Supplementary Figure 7: siRNA mediated knockdown of HSPA5/Bip and ATF6 in the U2OS cell line. A) The effects of two siRNAs targeting Hspa5/Bip (GNF250999 and GNF251000) are shown in red relative to control samples in blue and positive controls in green. B) Two further siRNAs were also validated in this screen (GNF251001 and GNF251002) with similar effects on amplitude. C) and D) show the effects of siRNA targeting ATF6. Dampening of circadian rhythm is shown for two of the four tested siRNAs. All traces are taken from http://biogps.org/circadian (Ref. 32).

## Slide 8
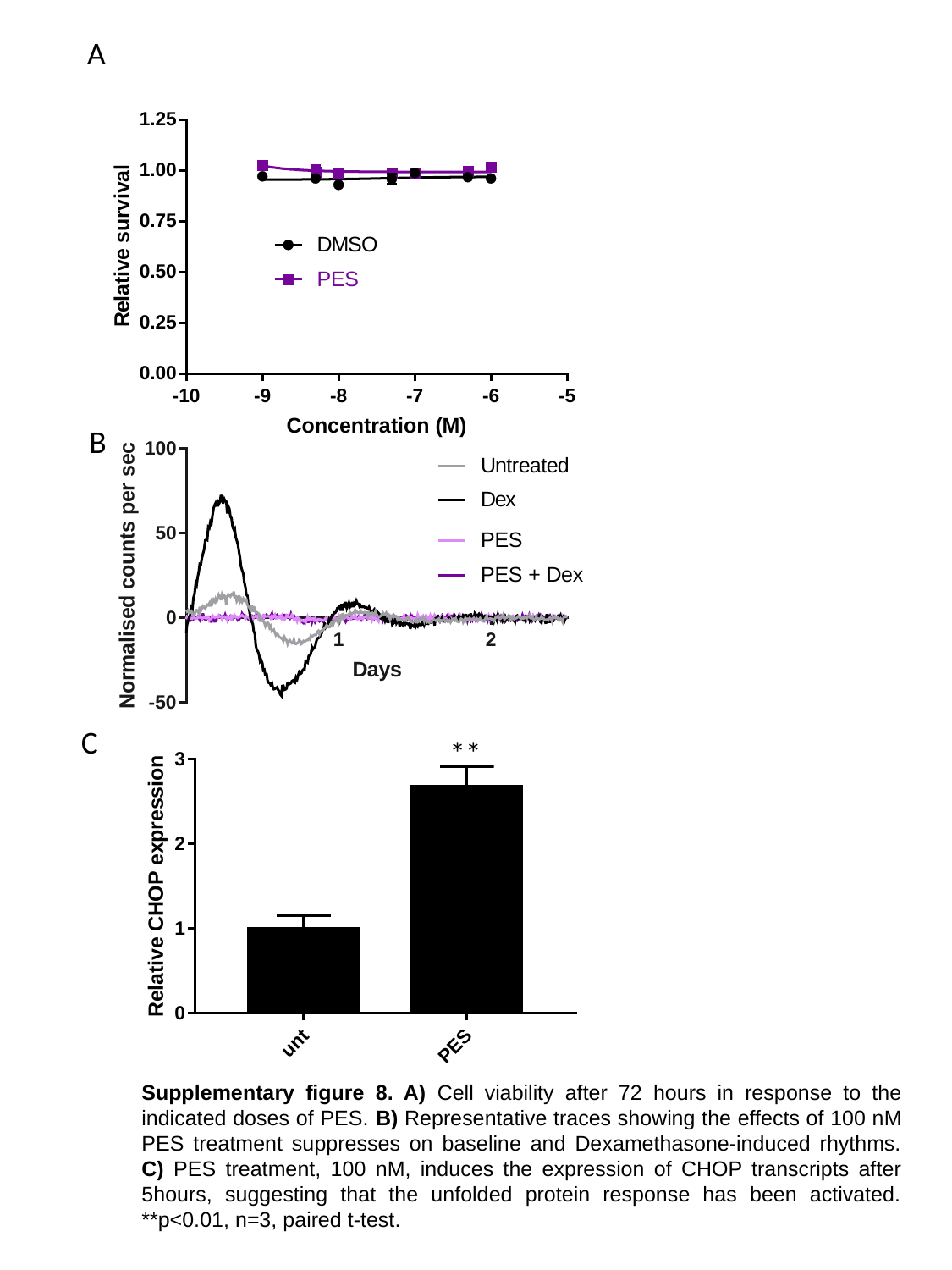

A
B
C
**
Supplementary figure 8. A) Cell viability after 72 hours in response to the indicated doses of PES. B) Representative traces showing the effects of 100 nM PES treatment suppresses on baseline and Dexamethasone-induced rhythms. C) PES treatment, 100 nM, induces the expression of CHOP transcripts after 5hours, suggesting that the unfolded protein response has been activated. **p<0.01, n=3, paired t-test.

## Slide 9
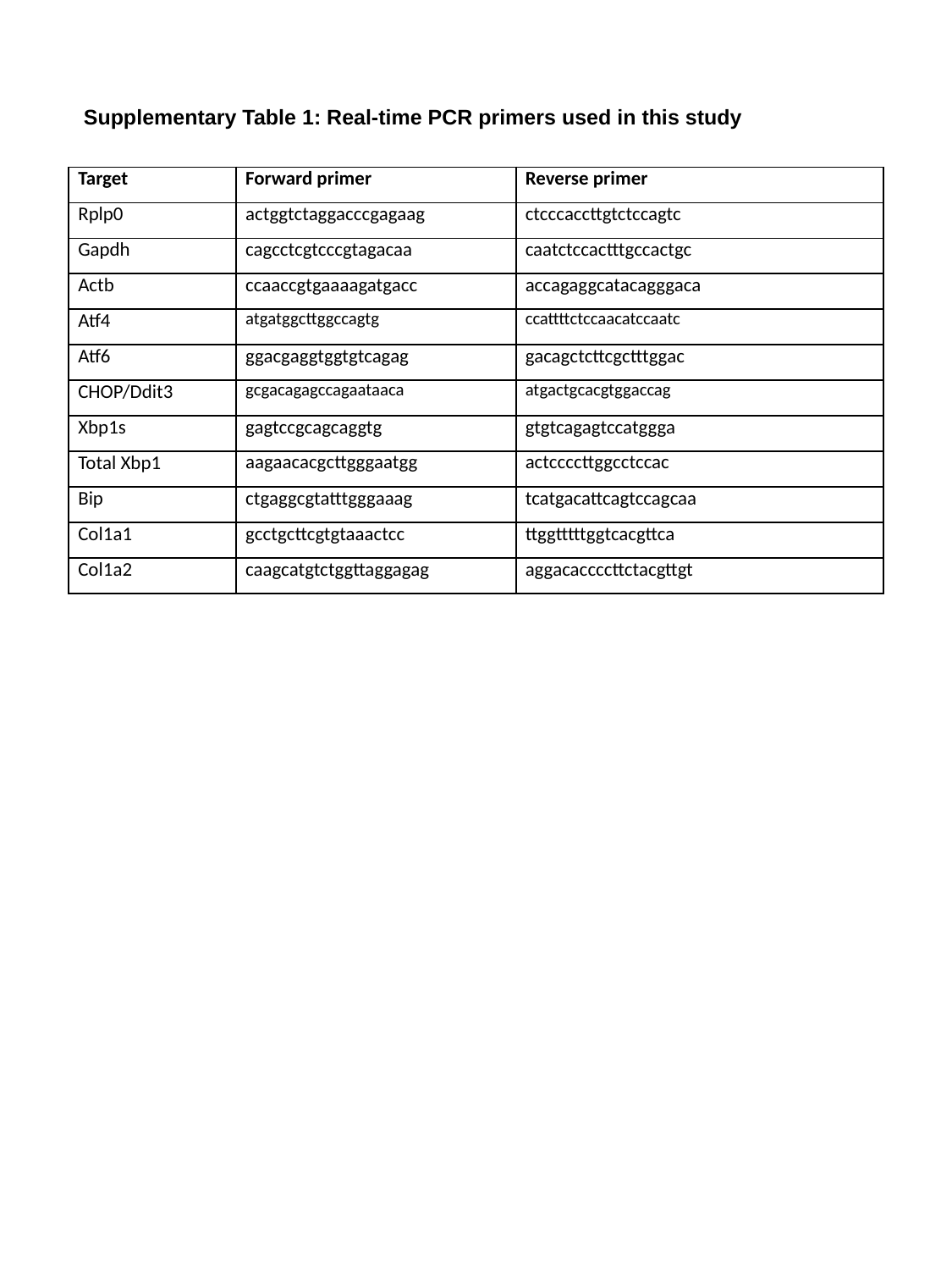

Supplementary Table 1: Real-time PCR primers used in this study
| Target | Forward primer | Reverse primer |
| --- | --- | --- |
| Rplp0 | actggtctaggacccgagaag | ctcccaccttgtctccagtc |
| Gapdh | cagcctcgtcccgtagacaa | caatctccactttgccactgc |
| Actb | ccaaccgtgaaaagatgacc | accagaggcatacagggaca |
| Atf4 | atgatggcttggccagtg | ccattttctccaacatccaatc |
| Atf6 | ggacgaggtggtgtcagag | gacagctcttcgctttggac |
| CHOP/Ddit3 | gcgacagagccagaataaca | atgactgcacgtggaccag |
| Xbp1s | gagtccgcagcaggtg | gtgtcagagtccatggga |
| Total Xbp1 | aagaacacgcttgggaatgg | actccccttggcctccac |
| Bip | ctgaggcgtatttgggaaag | tcatgacattcagtccagcaa |
| Col1a1 | gcctgcttcgtgtaaactcc | ttggtttttggtcacgttca |
| Col1a2 | caagcatgtctggttaggagag | aggacaccccttctacgttgt |
